# Calbindin Deficits May Underlie Dissociable Effects of 5-HT_6_ and mGlu_7_ Antagonists on Glutamate and Cognition in a Dual-Hit Neurodevelopmental Model for Schizophrenia

**DOI:** 10.1007/s12035-020-01938-x

**Published:** 2020-06-12

**Authors:** Sinead E. Shortall, Angus M. Brown, Eliot Newton-Mann, Erin Dawe-Lane, Chanelle Evans, Maxine Fowler, Madeleine V. King

**Affiliations:** School of Life Sciences, Medical School, Queen’s Medical Centre, The University of Nottingham, Nottingham, NG7 2UH UK

**Keywords:** Neonatal PCP, Isolation rearing, Glutamate, 5-HT_6_, mGlu_7_, Calbindin

## Abstract

Despite several compounds entering clinical trials for the negative and cognitive symptoms of schizophrenia, few have progressed beyond phase III. This is partly attributed to a need for improved preclinical models, to understand disease and enable predictive evaluation of novel therapeutics. To this end, one recent approach incorporates “dual-hit” neurodevelopmental insults like neonatal phencyclidine plus isolation rearing (PCP-Iso). Glutamatergic dysfunction contributes to schizophrenia pathophysiology and may represent a treatment target, so we used enzyme-based microsensors to evaluate basal- and drug-evoked glutamate release in hippocampal slices from rats that received neonatal PCP and/or isolation rearing. 5-HT_6_ antagonist-evoked glutamate release (thought to be mediated indirectly via GABAergic disinhibition) was reduced in PCP-Iso, as were cognitive effects of a 5-HT_6_ antagonist in a hippocampal glutamate-dependent novel object discrimination task. Yet mGlu_7_ antagonist-evoked glutamatergic and cognitive responses were spared. Immunohistochemical analyses suggest these findings (which mirror the apparent lack of clinical response to 5-HT_6_ antagonists in schizophrenia) are not due to reduced hippocampal 5-HT input in PCP-Iso, but may be explained by reduced calbindin expression. This calcium-binding protein is present in a subset of GABAergic interneurons receiving preferential 5-HT innervation and expressing 5-HT_6_ receptors. Its loss (in schizophrenia and PCP-Iso) would be expected to reduce interneuron firing and potentially prevent further 5-HT_6_ antagonist-mediated disinhibition, without impacting on responses of VIP-expressing interneurons to mGlu_7_ antagonism. This research highlights the importance of improved understanding for selection of appropriate preclinical models, especially where disease neurobiology impacts on cells mediating the effects of potential therapeutics.

**Electronic supplementary material:**

The online version of this article (10.1007/s12035-020-01938-x) contains supplementary material, which is available to authorized users.

## Introduction

Neurodevelopmental disorders like schizophrenia, attention deficit hyperactivity disorder, and autistic spectrum disorder have a complex etiology, involving combinations of early-life risk factors that trigger persistent long-term changes and disease emergence later in life [1]. Relatively poor management of negative and cognitive symptoms of schizophrenia by existing antipsychotics often prevents reintegration into society [2], and as a result, this disorder remains one of the top 10 causes of disability worldwide with an annual cost of over $158 billion in the USA alone [3]. 5-HT_6_ receptor antagonists and numerous other receptor- and transporter-selective compounds showed promising activity against seemingly relevant deficits in preclinical models, but disappointingly very few progressed beyond phase III clinical trials. This high attrition is partly attributed to a need for improved preclinical models, to further elucidate disease neurobiology and enable more predictive evaluation of novel therapeutics [4].

One approach to producing more comprehensive rodent models for neurodevelopmental disorders like schizophrenia involves “dual-hit” combinations of established perinatal and peripubertal interventions that each mirror different aspects of delayed symptom onset and multiple neurotransmitter involvement [5]. For example, neonatal NMDA receptor antagonist administration (between postnatal days 7–11 when sensitivity to their pro-apoptotic effects peaks [6]) followed by postweaning isolation rearing of gregarious rat pups induces more robust deficits than either manipulation alone [7–10]. Thus, combined neonatal phencyclidine (PCP) plus isolation rearing (PCP-Iso) produces more extensive cognitive impairment across a broader array of domains, including spatial reference and fear-motivated associative memory [7, 10], plus altered pro-social interaction and concomitant ultrasonic vocalizations [11, 12] that appear more akin to negative symptomatology than the increased aggression seen with single-hit isolation rearing [13]. These changes are accompanied by downregulation of hippocampal genes involved in glutamate metabolism, dopaminergic neurotransmission, and GABA receptor signaling, as well as those encoding parvalbumin and glutamic acid decarboxylase 67 (GAD_67_) [14]. Preliminary evidence suggests visual recognition memory deficits in the dual-hit model have some predictive validity, being reversed by the dopamine D_3_-preferring D_2_/D_3_ receptor partial agonist cariprazine [12] which is now approved by the FDA for management of schizophrenia [15], the atypical antipsychotic aripiprazole [12] that has modest cognitive benefit in some patients [16], and lamotrigine [14] which although not widely used may assist clozapine-resistant cases [17]. However, further insight into the molecular and neurochemical basis for differences between single and dual-hit models is essential to understand their potential utility in drug discovery for different patient subgroups or schizophrenia as a whole.

There is clear evidence for glutamatergic dysfunction in schizophrenia [18–22] and other disorders that feature cognitive impairment, and marked pharmaceutical interest in developing glutamate-based treatments [23]. Yet so far the possibility of more extensive glutamatergic deficits in combined versus separate neonatal PCP and isolation rearing models has not been studied at either a functional or protein expression level, and our initial experiment therefore focused on both these issues. We used enzyme-based microsensors to evaluate basal- and drug-evoked glutamate release in hippocampal slices from neonatal PCP-treated and/or isolation-reared rats, because this technique provides a direct method to selectively monitor extracellular glutamate on a second-by-second basis [24]. In addition, the ability to use separate slices from each individual to investigate a range of putative procognitive drugs that increase extracellular glutamate via different mechanisms (without the confounding influence of anesthesia required for magnetic resonance spectroscopy (MRS)) represents a marked contribution to the reduction component of the 3Rs initiative. Similar approaches have been applied to study epilepsy [25], spinal injury [26], and infection [27]. We focused on the hippocampus because glutamate hypofunction has been linked to declarative memory deficits in schizophrenia [28]. Although alterations within the dentate gyrus and CA3 are reported [29], we chose to record from CA1 due to evidence for volumetric and morphological abnormalities in early schizophrenia [30, 31], plus synaptic pathology in chronic cases [32]. We report that the expected glutamate release evoked by a 5-HT_6_ receptor antagonist was reduced in our dual-hit model, whereas glutamatergic responses to depolarization, reuptake inhibition, group III metabotropic receptor (mGlu) blockade, and mGlu_7_ allosteric antagonism all remained unaffected. Parallel western blot studies to investigate the underlying reasons for this focused on protein expression of vesicular glutamate transporters (VGLUT) 1–3 (required for presynaptic glutamate release), excitatory amino acid transporters (EAAT) 1–3 (responsible for glutamate reuptake), GAD_67_ (the GABA synthesis enzyme), vesicular GABA transporter (VGAT, required for presynaptic GABA release), plus 5-HT_6_ and mGlu_7_ receptors (that each regulate glutamate release via different mechanisms).

To determine the functional correlates of attenuated drug-evoked glutamate release in the slice preparation, our final experiment compared the cognitive-enhancing effects of 5-HT_6_ and mGlu_7_ antagonists in rats that received both PCP and isolation to those in single-hit isolation-only animals. We focused exclusively on novel object discrimination (NOD) because of its dependence on hippocampal glutamate [33–36], our previous findings with cariprazine, aripiprazole, and lamotrigine suggesting potential predictive validity of this test when combined with this model [12, 14], and the ability to perform repeated testing using a cross-over design to reduce animal numbers and further comply with the 3Rs initiative. Having noted a selective absence of 5-HT_6_ antagonist-mediated cognitive effects in the dual-hit model, we performed immunohistochemical analyses of 5-HT input to the hippocampus, plus the calbindin-positive cells that are its preferential target [37] and the main subset of 5-HT_6_ receptor-expressing GABAergic interneurons [38], in an attempt to provide mechanistic insight.

## Materials and Methods

### Animals

**Fig. 1:**
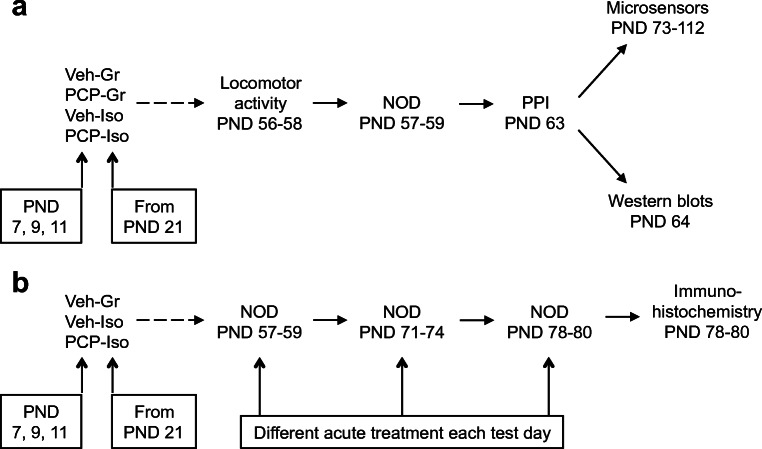
Summary of the experimental design. Two separate cohorts of male Lister hooded rats that received saline (1 ml/kg s.c.; Veh) or PCP (10 mg/kg) on PND 7, 9, and 11 were housed in social groups (Gr) or isolation (Iso) from weaning on PND 21. The first cohort **a** underwent locomotor activity, NOD, and PPI (*n* = 15–18 per treatment-housing combination) before balanced allocation to microsensor (*n* = 7–9) or western blot (*n* = 8–10) subgroups. The second cohort **b** underwent NOD on three occasions at 1–2-week intervals to receive acute vehicle (0.5% methylcellulose 1% Tween 80; 1 ml/kg i.p. 30 min before the familiarization trial), SB-399885 (10 mg/kg), or MMPIP (10 mg/kg) on separate test days in a pseudorandom order (*n* = 13–14 per neurodevelopmental condition), before tissue collection for immunohistochemistry

This research used a total of 113 male Lister-hooded rats (Charles River UK) maintained under controlled conditions (21 ± 2 °C, 55 ± 10% humidity, 12-h light-dark cycle; on at 07:00 h). For initial microsensor/western blot characterization of glutamatergic deficits, 69 pups from 14 litters were obtained with dams on postnatal day (PND) 3, randomized (by drawing lots) to receive saline vehicle (Veh; 1 ml/kg s.c.) or PCP HCl (10 mg/kg base) on PND 7, 9, and 11 [9], then housed in mixed treatment groups (3–4; Gr) or isolation from weaning on PND 21 (with allocation balanced across litters). Resultant Veh-Gr, PCP-Gr, Veh-Iso, and PCP-Iso were randomized (as above) to microsensor (*n* = 7–9) or western blot (*n* = 8–10) subgroups, with three age-matched drug-naïve group-housed rats (423–469 g; Charles River UK) for in-house microsensor validation. Subsequent NOD assessment of pharmacological sensitivity followed by immunohistochemistry used 41 pups from 6 litters (*n* = 13–14; Fig. 1).

Dams with litters were housed in individually ventilated cages with standard environmental enrichment. After weaning cages (Gr 32 × 51 cm, Iso 25 × 42 cm) contained only sawdust with grid lids to ensure maintenance of visual, olfactory, and auditory contact [39]. Handling was restricted to a single weekly cage change and body weight measurement until behavioral testing. Neonatal injections and behavioral testing occurred in the light phase (10:00–11:00 h and 09:00–16:00 h, respectively). All procedures were conducted in accordance with the Animals (Scientific Procedures) Act, 1986 and ARRIVE guidelines [40, 41], with University of Nottingham Local Ethical Committee approval. Group sizes were based on previous studies employing these techniques [12, 24, 42]. Data were obtained by trained observers unaware of neurodevelopmental history or any acute treatment.

### Microsensor and Western Blot Characterization of Glutamatergic Deficits

#### Prior Confirmation of Neonatal PCP and Isolation Rearing-Induced Behavioral Phenotype

To confirm expected development of the previously reported behavioral phenotype before tissue collection, rats underwent a short battery of tasks [14] selected for translational relevance to the positive and cognitive symptoms of schizophrenia [43–45] and which map to the arousal, cognitive, and sensorimotor sections of the Research Domain Criteria (RDoC) [46]. Tests were ordered from least to most aversive and are well established within the laboratory (e.g., [10, 12, 14, 47–49]) and described in detail elsewhere [42]. The length of time required to conduct microsensor recordings meant that more extensive behavioral evaluation including confirmation of previously reported deficits in fear-motivated associative memory [10, 12] was not possible, due to project license limits on the duration of isolation and weight restrictions for the required euthanasia method.

Locomotor activity [42, 47] was assessed (PND 56–58, with a balanced mix of housing and treatment groups each day) for 1 h in individual photobeam activity chambers (39 × 23.5 × 24.5 cm: San Diego instruments, CA, USA), where a single ambulation count was recorded for every two consecutive adjacent lower beam breaks, a single rearing count for every upper beam break, and a single fine movement count for each repeated lower beam break.

NOD [42, 48] was assessed the following day in the same arena. Rats received 3 min habituation, then two consecutive 3 min object exploration trials separated by 2 h. In the familiarization trial, rats encountered two identical bottles. For the choice trial, one was randomly replaced with a novel object (striped bottle). Object exploration (sniffing, licking, chewing, or having moving vibrissae while directing the nose towards and ≤ 1 cm) was timed and used to calculate the choice trial discrimination ratio (exploration of novel/total choice trial object exploration). The 2 h inter-trial interval was chosen to ensure intact memory in group-housed controls [48] and permit detection of neonatal PCP and/or isolation rearing-evoked deficits [10, 12, 14, 42]. However, we acknowledge this comparatively short interval makes the task less suitable for dissociating the effects of cognitive-enhancing test compounds on memory acquisition versus consolidation or retention, as can be performed with longer intervals [48].

Pre-pulse inhibition of the acoustic startle response (PPI [42, 49]) was assessed (PND 63) in SR-Lab startle response chambers (San Diego instruments, CA, USA) where rats received 5 min acclimatization to background white noise (62 dB), ten 120 dB startle trials, a pseudorandom mix of 50 trials with or without a preceding sub-threshold 72, 76, 80, or 84 dB prepulse, and then five final startle trials (all separated by 10–20 s). Individual whole-body startle responses were recorded for 100 ms after startle pulse onset and used to calculate area under the curve (AUC). For each trial type, the mean percentage PPI was calculated from mean AUC (after conditional elimination of values greater than two standard deviations from the mean, attributed to movement during startle delivery), using the equation % PPI = ((pulse alone AUC − prepulse AUC)/pulse alone AUC) × 100.

#### Glutamate Microsensor Recordings

**Fig. 2:**
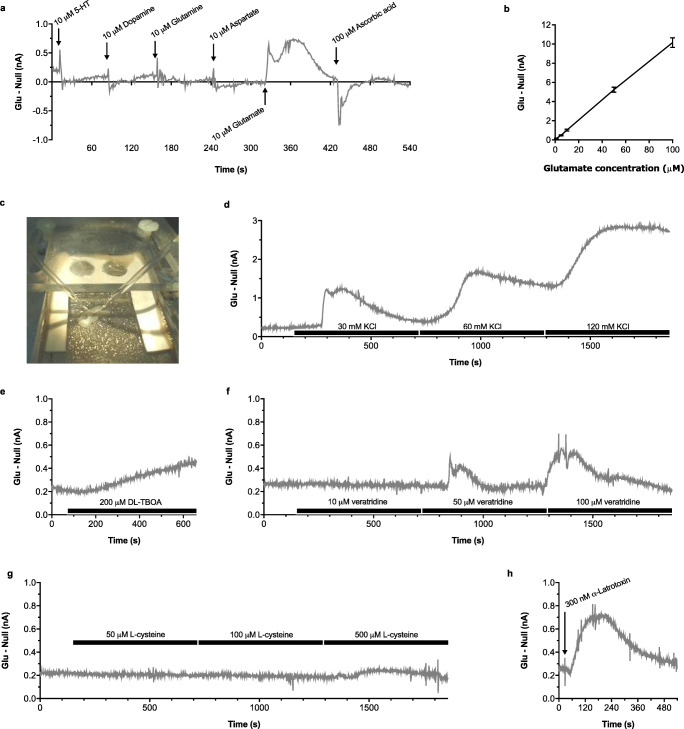
Characterization of the glutamate microsensor signal prior to studies in neurodevelopmentally manipulated rats. Representative **a**, **d**–**h** or mean ± SEM **b** difference in current output between glutamate and null sensors on addition of **a** exogenous glutamate or potential interfering agents, which demonstrates a selective response to glutamate, and **b** increasing concentrations of glutamate in the absence of tissue, which demonstrates a linear relationship, as well as following insertion of sensors into the CA1 region **c** of separate slices from drug-naïve group-housed rats (*n* = 3) where glutamate signals were increased by exposure to **d** elevated KCl, **e** the glutamate reuptake inhibitor DL-TBOA, **f** sodium channel activator veratridine, **g** antiport exchange substrate L-cysteine, or **h** α-latrotoxin which induces exocytosis from presynaptic vesicles, suggesting neuronal origin of the glutamate signal. Bars represent 10-min perfusion via the aCSF reservoir, and arrows local application to the interface chamber

Methods were modified from Oldenziel et al. [24]. Rats were euthanized by cervical dislocation and brains immersed in ice-cold artificial cerebrospinal fluid (aCSF; 126 mM NaCl, 3.0 mM KCl, 2.0 mM CaCl_2_, 2.0 mM MgCl_2_, 1.2 mM NaH_2_PO_4_, 26 mM NaHCO_3_, and 10 mM glucose) were gassed with 95% O_2_/5% CO_2_. Transverse hippocampal slices (400 μm) prepared using a vibrating microtome (Leica Biosystems, Nussloch, Germany) recovered for at least 1 h at room temperature in a brain slice prechamber (Harvard Apparatus, Cambridge, UK). Glutamate and null biosensors (50 μm diameter × 500 μm length: Sarissa Biomedical Ltd., Coventry, UK [50]) were rehydrated (100 mM NaCl, 1 mM MgCl_2_, 2 mM glycerol, 10 mM NaPi, pH 7.4 for 10 min then glycerol for 5 min), polarized (MicroC: WPI, Sarasota, FL, USA) to 500 mV versus an Ag/AgCl reference electrode in a brain slice interface chamber (Harvard Apparatus), cycled from − 500 to 500 mV at 100 mV/s for 10 cycles, and then checked for initial sensitivity to 10 μM L-glutamate (response > 0.1 nA). Test solutions and potential interfering agents (10 μM 5-HT, dopamine, glutamine, aspartate, and 100 μM ascorbic acid) were also examined in the absence of tissue. Signals were recorded using Clampex 9.2 (Molecular Devices, Wokingham, UK). Individual slices were transferred to the interface chamber where they were superfused with aCSF containing 2 mM glycerol (37 °C) and continuously aerated by humidified 95% O_2_/5% CO_2_. Glutamate and null sensors were inserted in close proximity (~ 200 μm; V-shaped form) into CA1 (Fig. 2c) and equilibrated for 30 min.

Validation studies in tissue from drug-naïve group-housed animals examined stimulation of glutamate by KCl depolarization (30, 60, 120 mM, with corresponding reductions in NaCl to maintain osmotic balance), the glutamate reuptake inhibitor DL-TBOA (200 μM), sodium channel activator veratridine (10, 50, 100 μM), antiport exchange substrate L-cysteine (50, 100, 500 μM), and α-latrotoxin (300 nM) which induces exocytosis from presynaptic vesicles, plus inhibition of basal-, KCl-, and TBOA-evoked release by the voltage-dependent sodium channel blocker tetrodotoxin (TTX; 20 μM). Responses to KCl depolarization, TBOA inhibition of glutamate reuptake, the 5-HT_6_ antagonist SB-399885 (3 μM ± 120 mM KCl), group III mGlu antagonist CPPG (100 μM ± 120 mM KCl), and mGlu_7_ allosteric antagonist MMPIP (100 μM ± 120 mM KCl) were then compared in slices from the four different neurodevelopmental conditions (PND 73–112). Antagonist concentrations were similar to previous in vitro [51, 52] and hippocampal slice [53, 54] studies, and intentionally higher than achieved in vivo to allow for slice penetration [55]. Compounds (one per slice) were perfused via the aCSF reservoir (10 min or 15 min for 5-HT_6_/mGlu antagonists with high KCl) or applied direct to slices (α-latrotoxin). Point calibration to 10 μM L-glutamate was repeated postslice. The difference in current output (nA) between glutamate and null sensors was calculated off-line and used with calibration data to determine glutamate concentration. An average basal extracellular glutamate for each animal was derived from the five separate slices used to examine AUC responses to different glutamatergic manipulations, such that the final *n* for each neurodevelopmental condition represents the number of different animals each investigated with different pair of glutamate and null sensors.

#### Western Blots

Rats were euthanized on PND 64 by concussion and immediate decapitation, and western blotting was performed as previously described [42]. Portions of nitrocellulose membranes containing proteins > 40 kDa were incubated overnight (4 °C) with mouse monoclonal or rabbit polyclonal primary antibodies against VGLUT1, VGLUT2 (1:500; Merck Millipore, Watford, UK), VGLUT3 (1:500; Abcam, Cambridge, UK), EAAT1, EAAT2, EAAT3 (1:500; Alpha Diagnostic International, San Antonio, TX, USA), GAD_67_ (1:1000; Merck Millipore), VGAT (1:400; Abcam), 5-HT_6_ (1:500; Abcam), or mGlu_7_ (1:500; Merck Millipore). Corresponding portions containing proteins < 40 kDa were incubated with mouse monoclonal or rabbit polyclonal primary antibodies against the housekeeping protein GAPDH (1:20,000; Sigma-Aldrich, Poole, UK). After infrared secondary antibody (800 CW anti-mouse or anti-rabbit, 1:10,000; LI-COR, Cambridge, UK) incubation (1 h in the dark), bands were detected and quantified using a LI-COR Odyssey system and data expressed as a percentage of GAPDH.

### NOD Assessment of Pharmacological Sensitivity, Followed by Immunohistochemistry

#### NOD

Consistent with previous observations [10], behavioral phenotyping of our microsensor/western blot cohort found no effect of single-hit neonatal PCP on NOD (Fig. S1d-e), so to comply with the 3Rs principle, we did not include a PCP-Gr subgroup (with predicted intact NOD) in the cohort comparing pharmacological reversal of NOD deficits in Veh-Iso versus PCP-Iso. Veh-Gr, Veh-Iso, and PCP-Iso underwent NOD (as described above) on three occasions at 1–2 week intervals (PND 57–80) to receive vehicle (0.5% methylcellulose 1% Tween 80; 1 ml/kg i.p. 30 min before familiarization), SB-399885 (10 mg/kg), or MMPIP (10 mg/kg) on separate days in a pseudorandom order and serve as their own controls. SB-399885 and MMPIP doses were selected from studies demonstrating behavioral activity, including reversal of NOD deficits in other models for schizophrenia [42, 56], without motor impairment [57–59].

#### Immunohistochemistry

Rats were euthanized after the final NOD (PND 78–80) by concussion and immediate decapitation. Brain hemispheres were immersed fixed in 4% paraformaldehyde then 30% sucrose (each overnight, 4 °C) and frozen in isopentane on dry ice. Serial coronal sections (60 μm) were obtained throughout the dorsal hippocampus using a freezing microtome (Anglia Scientific, Cambridge, UK) and stored in 30% glycerol, 30% ethylene glycol (− 20 °C) until free-floating immunohistochemistry. One PCP-Iso was excluded from the rest of the study due to technical difficulties during slicing. Six evenly spaced sections were selected from each rat, to span approximately Bregma − 2.44 to − 4.42 according to a digital atlas [60]. Sections were washed (4 × 5 min) in phosphate-buffered saline (PBS), incubated (1 h) in 2% goat or donkey serum in buffer 1 (0.5% BSA, 0.3% Triton X-100), then (overnight, 4 °C) rabbit or goat polyclonal primary antibodies against calbindin or 5-HT (Abcam: 1:500 in buffer 1), or buffer alone for negative control. Sections were washed (3 × 5 min) in buffer 2 (0.15% BSA, 0.1% Triton X-100), incubated (1 h in the dark) in Alexa Fluor 568 goat anti-rabbit or 594 donkey anti-goat secondary antibodies (Thermo-Fisher, Loughborough, UK: 1:500 in buffer 2), washed (2 × 5 min each) in buffer 2 and PBS, and then mounted on gelatin-subbed slides and air-dried. They were then rinsed with PBS, counterstained with DAPI nuclear stain (Sigma-Aldrich: 1:2000 in H_2_O; 30 s), rinsed with H_2_O, and cover slipped with DABCO (Sigma-Aldrich: 0.2% in 90% glycerol) then stored at 4 °C. With the expectation of DAPI, all solutions were prepared in PBS.

Sections were viewed on a Nikon EFD-3 fluorescence microscope and images obtained using an Insight QE camera and SPOT Imaging software (Diagnostic Instruments Inc., MI, USA). The number of calbindin-positive cells within strata oriens, radiatum, and lacunosum-moleculare of the subiculum/fasciola cinereum, CA1, and CA2/3 was counted from × 4 images manually reconstructed to cover the entire dorsal hippocampus. The intensity of calbindin and 5-HT immunoreactivity in consistently placed × 20 images of CA1 (encompassing strata oriens, pyramidale, and radiatum; Fig. 5a), and of 5-HT immunoreactivity in further × 40 images entirely within strata oriens or radiatum (Fig. 5e), was quantified using Fiji [61]. Anatomical boundaries were determined using a digital atlas [60].

### Drugs

PCP HCl, L-glutamate, 5-HT, dopamine, ascorbic acid, and L-cysteine were purchased from Sigma-Aldrich, α-latrotoxin from Enzo Life Sciences (Exeter, UK), and all other compounds from Tocris (Bristol, UK). For microsensor studies, stock solutions of TBOA, SB-399885, CPPG, MMPIP, TTX (2–10 mM in saline), and α-latrotoxin (300 nM in 50% glycerol) were stored in aliquots at − 20 °C and diluted in aCSF on the day of use. All other solutions were prepared daily.

### Statistical Analysis

All analyses were performed using GraphPad Prism (v7) or IBM SPSS (v24). After confirming normality and homogeneity of variance, data from the microsensor/western blot cohort were analyzed by three-way repeated measures ANOVA (with time, object, prepulse, applied drug concentration, or glutamate transporter subtype as a within-subject factor) or two-way ANOVA (total locomotor activity, NOD discrimination ratio, microsensor responses to single-drug concentrations, and remaining protein expression) with neonatal treatment and housing as between-subject factors. Data from the pharmacological NOD and immunohistochemistry cohort were analyzed by three-way repeated measures ANOVA with acute treatment and object, or hippocampal subfield and cell layer as within-subject factors and neurodevelopmental condition as a between-subject factor. NOD discrimination ratios were analyzed by two-way repeated measures ANOVA with acute treatment as a within-subject factor, and remaining immunohistochemical data by one-way ANOVA, with neurodevelopmental condition as a between-subject factor in each case. Pearson’s *r* correlation analyses were also performed between immunohistochemical and discrimination ratio data. Post hoc within- and between-subject comparisons used Sidak and Tukey tests, respectively. *P* < 0.05 was regarded as statistically significant and data are presented as mean ± SEM.

## Results

### Microsensor and Western Blot Characterization of Glutamatergic Deficits

#### Prior Confirmation of Neonatal PCP and Isolation Rearing-Induced Behavioral Phenotype

The time course of ambulation in a novel arena showed a time × housing interaction (*F*_(11,605)_ = 3.055; *P* < 0.001), and although there were no time × treatment or time × treatment × housing interactions, PCP-Iso had higher ambulation than Veh-Gr controls and single-hit PCP-Gr or Veh-Iso at multiple time points (Fig. S1a). Total ambulation showed treatment (*F*_(1,55)_ = 3.872; *P* < 0.05) and housing (*F*_(1,55)_ = 3.817; *P* < 0.05) effects and was higher in PCP-Iso (but not Veh-Iso or PCP-Gr) than in Veh-Gr (*P* < 0.05; Fig. S1b). Similar patterns were observed for rearing and fine movement (data not shown).

NOD testing confirmed an effect of object (*F*_(1,65)_ = 37.016; *P* < 0.001) and an object × housing interaction (*F*_(1,65)_ = 18.979, *P* < 0.001) during the choice trial. Veh-Gr and PCP-Gr were both able to discriminate the novel from familiar object (*P* < 0.0001 and *P* < 0.001, respectively), but Veh-Iso and PCP-Iso were not (*P* > 0.05; Fig. S1d). This isolation-induced impairment was further supported by a housing effect on the discrimination ratio (*F*_(1,65)_ = 22.37, *P* < 0.0001), which was lower in both Veh-Iso (*P* < 0.001) and PCP-Iso (*P* < 0.01) than in Veh-Gr (Fig. S1e). Importantly, these changes occurred without any spatial preference between identical objects during the familiarization trial (Fig. S1c) or any differences in total object exploration (data not shown).

PPI testing showed the expected effect of prepulse volume (*F*_(2,130)_ = 122.534; *P* < 0.001), although the prepulse × housing interaction just failed to reach statistical significance (*F*_(2,130)_ = 2.921, *P* = 0.057; Fig. S1f). There were no differences in startle reactivity (data not shown).

Taken together, these findings suggest the current PCP-Iso cohort had slightly more marked locomotor hyperactivity than previous studies, and the expected robust NOD impairment, but a smaller PPI deficit than when these tests were performed after an extra week of isolation [10, 14], which was not compatible with the current microsensor study design. We therefore progressed these animals to planned microsensor and western blot studies, given the novelty of these proposed ex vivo measures following isolation rearing.

#### Effect of Neonatal PCP and Isolation Rearing on Glutamate Release from Hippocampal Slices

Validation studies confirmed that in the absence of tissue, sensors responded to exogenous glutamate (Fig. 2a) in a linear manner (Fig. 2b), but not to test compounds (data not shown) or potential interfering agents (Fig. 2a). Basal extracellular glutamate in slices from drug-naïve group-housed rats (2.43 ± 0.52 μM) was reduced by TTX (1.40 ± 0.27 μM; − 42%; *P* < 0.05) and elevated by KCl (Fig. 2d), TBOA (Fig. 2e), veratridine (Fig. 2f), L-cysteine (Fig. 2g), and α-latrotoxin (Fig. 2h). KCl- and TBOA-evoked responses were both sensitive to TTX (data not shown).

**Fig. 3:**
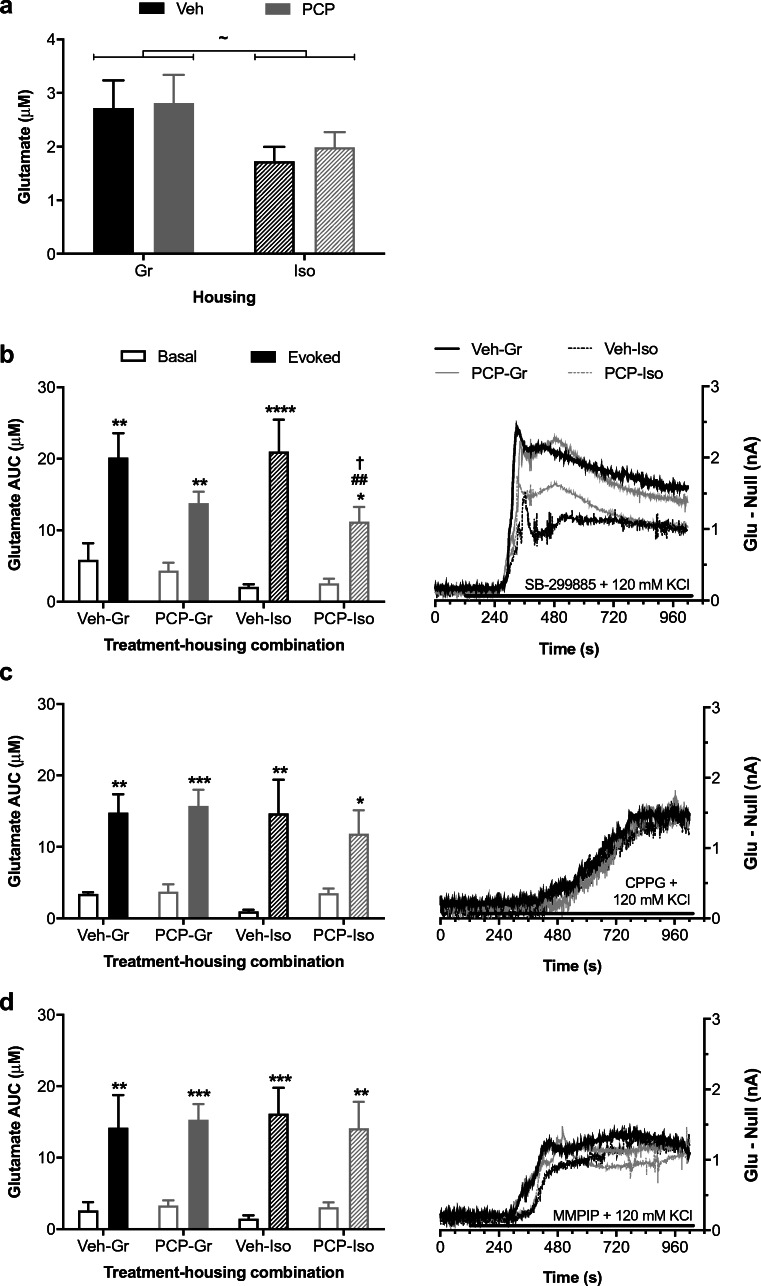
Effect of neonatal PCP and isolation rearing on glutamate release from hippocampal slices. Mean ± SEM (left-hand *y*-axis) **a** basal extracellular glutamate concentration and evoked AUC responses to **b** 3 μM SB-399885, **c** 100 μM CPPG, and **d** 100 μM MMPIP in the presence of 120 mM KCl for 15 min, together with (**b–d** right-hand *y*-axis) representative difference in current output between glutamate and null sensors. Bars represent 15-min perfusion via the aCSF reservoir. Male Lister hooded rats that received saline (1 ml/kg s.c.; Veh) or PCP (10 mg/kg) on PND 7, 9, and 11 were housed in groups (Gr) or isolation (Iso) from weaning on PND 21, with hippocampal slices obtained on PND 73–112 (*n* = 7–9 per treatment-housing combination). There were main effects of **a** isolation on basal extracellular glutamate levels (*P* < 0.05), and glutamate release was evoked by the combination of elevated KCl with **b** SB-399885 (*P* < 0.001), **c** CPPG (*P* < 0.001), or **d** MMPIP (*P* < 0.001). Of note, the release evoked by **b** SB-399885 plus high KCl interacted with neonatal PCP treatment (*P* < 0.05) and was reduced in PCP-Iso compared to Veh-Gr or Veh-Iso, whereas release evoked by **c** CPPG plus high KCl or **d** MMPIP plus high KCl remained unaffected by neonatal PCP treatment or isolation rearing. ^~^*P* < 0.05 versus Gr (two-way ANOVA); *P < 0.05; **P < 0.01; ***P < 0.001; ****P < 0.0001 versus basal in the same slice (three-way repeated measures ANOVA with Sidak post hoc); ^†^*P* < 0.05 versus Veh-Gr; ^##^*P* < 0.01 versus Veh-Iso (two-way ANOVA with Tukey post hoc)

Slices from neurodevelopmentally manipulated rats revealed a main effect of housing on basal hippocampal glutamate (*F*_(1,28)_ = 4.567; *P* < 0.05) which was 32% lower in Iso than Gr (Fig. 3a) but did not reach post hoc significance for Veh-Iso or PCP-Iso compared to Veh-Gr or PCP-Gr. KCl-evoked (*F*_(2,56)_ = 21.834; *P* < 0.001) and TBOA-evoked (*F*_(1,28)_ = 15.665; *P* < 0.001) increases were both unaffected by neurodevelopmental history (data not shown). Neither SB-399885, CPPG, nor MMPIP influenced extracellular glutamate levels when administered alone (data not shown), but of particular note, the release evoked by the 5-HT_6_ antagonist in the presence of KCl (*F*_(1,28)_ = 71.528; *P* < 0.001) interacted with neonatal PCP treatment (*F*_(1,28)_ = 6.213; *P* < 0.05) and was attenuated in PCP-Iso compared to both Veh-Gr (*P* < 0.05) and Veh-Iso (*P* < 0.01; Fig. 3b). No such reduction was seen for release evoked by the group III mGlu antagonist (*F*_(1,28)_ = 56.539; *P* < 0.001; Fig. 3c) or mGlu_7_ allosteric antagonist (*F*_(1,28)_ = 66.418; *P* < 0.001; Fig. 3d) with KCl, which remained unaffected by treatment or housing.

#### Effect of Neonatal PCP and Isolation Rearing on Hippocampal Expression of Glutamatergic and GABAergic Markers, plus 5-HT_6_ and mGlu_7_ Receptors

Protein expression data are shown in Fig. S2 and Fig. S3. There was a subtype × treatment × housing interaction for VGLUT expression (*F*_(2,56)_ = 4.306; *P* < 0.05). Despite no treatment × housing interaction for VGLUT1 (*F*_(1,32)_ = 3.095; *P* = 0.0881; Fig. S2a), there were interactions for the ratio of VGLUT1:VGLUT2 (*F*_(1,32)_ = 5.473; *P* < 0.05; Fig. S3a) and VGLUT1 as a proportion of total VGLUT (*F*_(1,32)_ = 4.952; *P* < 0.05; Fig. S3b), which were lower in PCP-Iso than in PCP-Gr (*P* < 0.05; − 35% and − 22% respectively). There was no subtype × treatment × housing interaction for EAATs, but a treatment × housing interaction for EAAT2 (*F*_(1,32)_ = 4.303; *P* < 0.05) which was lower in PCP-Iso than in Veh-Iso (*P* < 0.05, − 20%; Fig. S2e). There was also a treatment × housing interaction for 5-HT_6_ expression (*F*_(1,32)_ = 5.127; *P* < 0.05; Fig. S2i) but between-group differences did not reach post hoc significance.

### NOD Assessment of Pharmacological Sensitivity, Followed by Immunohistochemistry

#### Effect of Neonatal PCP and Isolation Rearing on the Cognitive Effects of 5-HT_6_ and mGlu_7_ Receptor Antagonists in the NOD Task

**Fig. 4:**
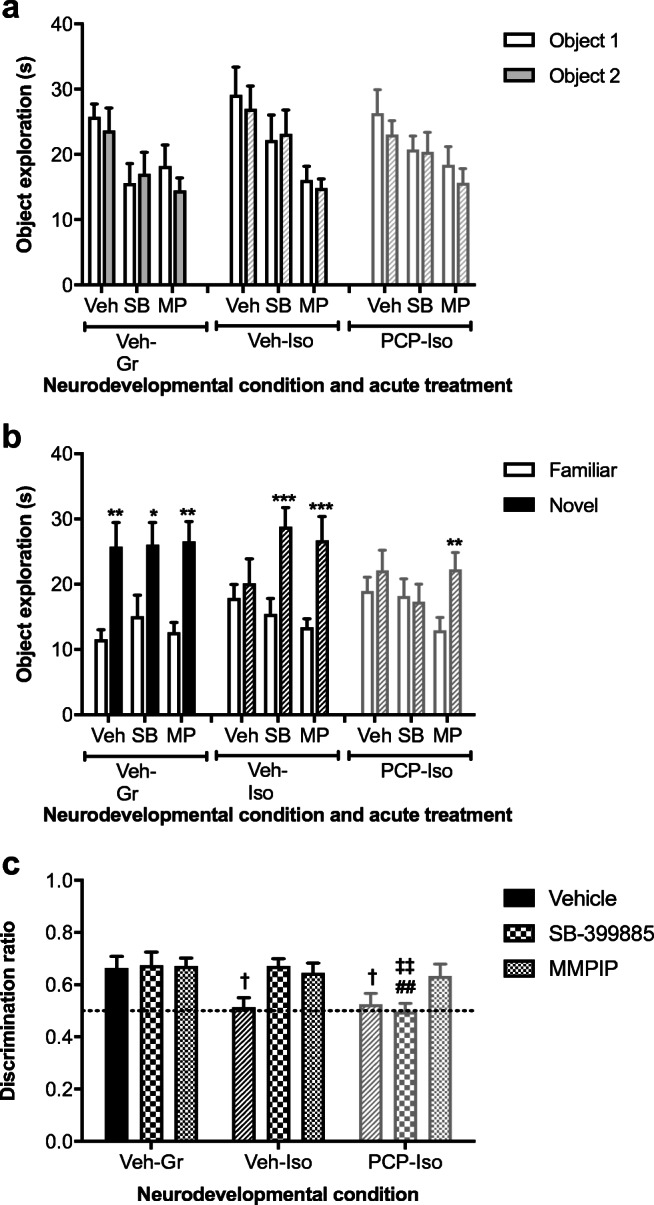
Impact of combined neonatal PCP and isolation rearing on the ability of a 5-HT_6_ (but not mGlu_7_) antagonist to reverse isolation-induced cognitive deficits in the NOD task. Mean ± SEM time spent exploring **a** two identical objects during the familiarization trial and **b** the novel and familiar object during the choice trial 2 h later, as well as **c** choice trial discrimination ratio (time exploring novel/total choice trial object exploration). Male Lister hooded rats that received saline (1 ml/kg s.c.; Veh) or PCP (10 mg/kg) on PND 7, 9, and 11 were housed in groups (Gr; Veh only) or isolation (Iso; Veh and PCP) from weaning on PND 21, then underwent NOD on three occasions at 1–2-week intervals (PND 57–80) to receive acute vehicle (Veh; 0.5% methylcellulose 1% Tween 80; 1 ml/kg i.p. 30 min before the familiarization trial), SB-399885 (SB; 10 mg/kg), or MMPIP (MP; 10 mg/kg) on separate test days in a pseudorandom order and serve as their own controls (*n* = 13–14 per neurodevelopmental condition). In the familiarization trial **a**, there was a main effect of acute treatment on the duration of object exploration (*P* < 0.001), which was decreased by SB-399885 and MMPIP, but importantly, no spatial preference between identical objects or any neurodevelopmental condition × treatment interaction. In the choice trial **b**, there was an effect of object (*P* < 0.001) and an object × neurodevelopmental condition interaction (*P* < 0.01). Veh-Gr discriminated the novel object following acute vehicle and this intact memory was not further enhanced by acute treatment, consistent with a ceiling effect. Impaired memory was restored by SB-399885 in single-hit Veh-Iso but not dual-hit PCP-Iso, whereas MMPIP remained effective in both models. Accordingly, discrimination ratios (**c**) were lower in Veh-Iso and PCP-Iso than in Veh-Gr following acute vehicle, and of note also lower in PCP-Iso than the other two neurodevelopmental conditions following acute SB-399885. *P < 0.05; **P < 0.01; ***P < 0.001 versus the familiar object following the same acute treatment in the same rats (three-way repeated measures ANOVA with Sidak post hoc); ^†^*P* < 0.05 versus acute vehicle in Veh-Gr, ^‡‡^*P* < 0.01 versus acute SB-399885 in Veh-Gr; ^##^*P* < 0.01 versus acute SB-399885 in Veh-Iso (two-way ANOVA with Tukey post hoc)

There was an acute treatment effect on the duration of familiarization trial object exploration (*F*_(2,76)_ = 12.684; *P* < 0.001) which was decreased by SB-399885 and MMPIP, but importantly no spatial preference between identical objects nor any neurodevelopmental condition × acute treatment interaction (Fig. 4a). In contrast, there were choice trial effects of object (*F*_(1,38)_ = 69.167; *P* < 0.001) plus an object × neurodevelopmental condition interaction (*F*_(2,38)_ = 6.382, *P* < 0.01). Veh-Gr discriminated the novel from familiar object following acute vehicle (*P* < 0.01), inferring intact memory, and this was not modified by SB-399885 (*P* < 0.05) or MMPIP (*P* < 0.01), consistent with a ceiling effect. Veh-Iso and PCP-Iso were both unable to discriminate following acute vehicle (*P* > 0.05), suggesting an inability to remember experiences from the familiarization trial 2 h previously. Memory was restored by SB-399885 in single-hit Veh-Iso (*P* < 0.001) but not dual-hit PCP-Iso (*P* > 0.05), whereas MMPIP remained effective in both models (*P* < 0.001 and *P* < 0.01, respectively; Fig. 4b). Discrimination ratios support this pattern, being lower in both Veh-Iso and PCP-Iso than in Veh-Gr following acute vehicle (*P* < 0.05), and of particular note also lower in PCP-Iso than the two other neurodevelopmental conditions following acute SB-399885 (*P* < 0.01; Fig. 4c).

#### Effect of Neonatal PCP and Isolation Rearing on Hippocampal Calbindin and 5-HT Immunoreactivity

**Fig. 5:**
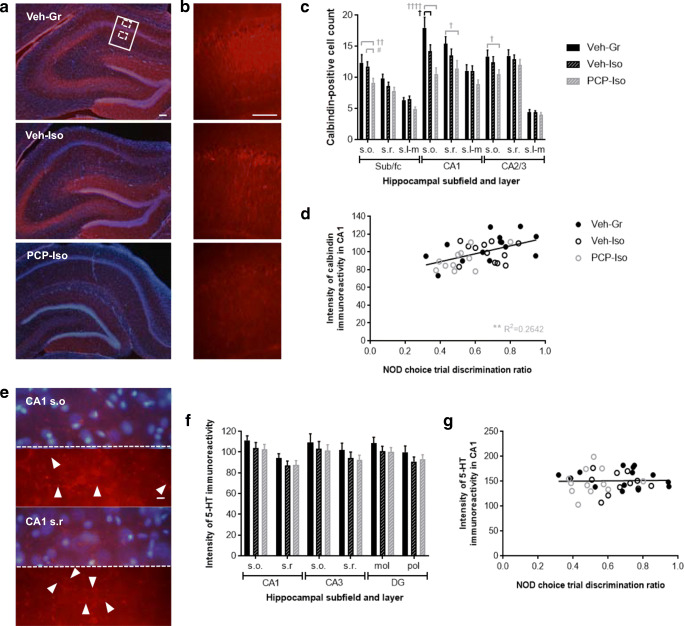
Impact of combined neonatal PCP and isolation rearing on hippocampal calbindin and 5-HT immunoreactivity in the dorsal hippocampus. Representative **a**, **b** calbindin immunoreactivity throughout **a** all subfields and **b** part of CA1 (indicated in **a** by a solid border), together with **e** higher magnification images of typical 5-HT immunoreactivity in CA1 strata oriens (s.o.) and radiatum (s.r.) locations (indicated in **a** by dashed borders). Red represents **a**, **b** calbindin or **e** 5-HT, and blue in **a** and the top portion of each image in **e** represents DAPI nuclear counterstain; the bottom portion of each image in **e** is a duplicate presented without the nuclear counterstain. Scale bars are equivalent to 100 μm **a**, **b** or 10 μm **e**. Mean ± SEM **c** number of calbindin-positive cells in s.o., s.r., and stratum lacunosum-moleculare (s.l-m) of the dorsal hippocampal subiculum/fasciola cinereum (sub/fc), CA1, and CA2/3, and **f** intensity of 5-HT immunofluorescence in s.o. and s.r. of dorsal hippocampal CA1 and CA3 and molecular (mol) and polymorphic (pol) layers of the dentate gyrus (DG), plus correlation analyses of **d** calbindin and **g** 5-HT immunofluorescence intensity in CA1 versus the NOD choice trial discrimination ratio (time exploring novel/total choice trial object exploration) following acute SB-399885. Male Lister hooded rats that received saline (1 ml/kg s.c.; Veh) or PCP (10 mg/kg) on PND 7, 9, and 11 were housed in groups (Gr; Veh only) or isolation (Iso; Veh and PCP) from weaning on PND 21, then underwent NOD on three occasions (PND 57–80) before tissue collection (PND 78–80), to receive vehicle (0.5% methylcellulose 1% Tween 80; 1 ml/kg i.p. 30 min before the familiarization trial), SB-399885 (10 mg/kg), or MMPIP (10 mg/kg) on separate test days in a pseudorandom order and serve as their own controls (*n* = 13–14 per neurodevelopmental condition). Calbindin immunoreactivity **a** was strongest in the dentate gyrus and stratum pyramidale, and remaining cells matched the distribution of GABA interneurons. The **b** intensity of calbindin immunoreactivity in CA1 and **c** number of calbindin-positive cells throughout dorsal hippocampal subfields and cell layers were both influenced by neurodevelopmental condition (*P* < 0.05 and *P* < 0.01, respectively), and although there were some reductions in single-hit Veh-Iso, these were much more extensive in PCP-Iso and correlated with **d** NOD performance following acute administration of SB-399885. Patterns of **e** 5-HT immunoreactivity were consistent with labeling of fine varicose axons (marked by arrowheads) but labeling intensity **f** was not influenced by neurodevelopmental condition and **g** did not correlate with NOD flowing SB-399885 administration. ^†^*P* < 0.05 Veh-Iso and ^†^*P* < 0.05; ^††^*P* < 0.01; ^††††^*P* < 0.0001 PCP-Iso versus Veh-Gr; ^#^*P* < 0.05 PCP-Iso versus Veh-Iso (three-way repeated measures ANOVA with Tukey post hoc)

There was typical intense calbindin labeling in the dentate gyrus and stratum pyramidale, which prevented cell counting within these regions. Remaining cells matched the distribution of GABA interneurons [62] (Fig. 5a, b) and their counts were influenced by neurodevelopmental history (*F*_(2,37)_ = 6.795; *P* < 0.01) and reduced in PCP-Iso (80 ± 3) compared to Veh-Gr (101 ± 5; *P* < 0.01) or Veh-Iso (95 ± 3; *P* < 0.05). This was predominantly due to changes in CA1 (PCP-Iso versus Veh-Gr *P* < 0.0001 and Veh-Iso *P* < 0.05), where labeling intensity was also affected by neurodevelopmental history (*F*_(2,37)_ = 3.294; *P* < 0.05) and lower in PCP-Iso (91 ± 4) than in Veh-Gr (105 ± 4; *P* < 0.05). Reduced counts in Veh-Iso were restricted to strata oriens of CA1 but extended in PCP-Iso to strata radiatum, plus strata oriens of remaining subfields (Fig. 5c). Significant positive correlations were observed between calbindin immunoreactivity and NOD discrimination ratios when rats received SB-399885 (Fig. 5d), but there was no link between calbindin immunoreactivity and cognitive performance when the same rats received either vehicle or MMPIP (data not shown).

Labeling of varicose 5-HT axons (Fig. 5e, which are known to preferentially contact calbindin-positive interneurons [37, 63]), showed the expected effect of cell layer [64] but was not influenced by neurodevelopmental history (Fig. 5f) and did not correlate with NOD following SB-399885 (Fig. 5g) or any other acute treatment (data not shown).

## Discussion

Combining two established rodent neurodevelopmental models for schizophrenia, neonatal PCP and isolation rearing, produces a wider range of behavioral and neurochemical alterations akin to the core pathophysiology of schizophrenia than either alone [7, 10–12]. Our locomotor data again support this more pronounced change in PCP-Iso, and the reliable NOD impairment in 100% of 9 PCP-Iso cohorts now examined in our laboratory confirm this deficit in visual recognition memory is more reproducible than the reported 70% following single-hit isolation [39]. PPI deficits seen in two previous PCP-Iso cohorts [10, 14] were not observed here, in only the third cohort to undergo this test, perhaps due to the slightly earlier timing of this assessment to accommodate subsequent microsensor studies while remaining within project license limits on the duration of isolation and weight restrictions for the required euthanasia method. Nevertheless, preliminary suggestions of a PPI deficit in 67% of the three PCP-Iso cohorts examined to date may represent some improvement on the approximately 55% of 18 single-hit isolate cohorts examined within our laboratory, particularly if the PCP-Iso deficit can be maximized by closely controlling timing of the assessment. Although assessment of fear-motivated memory could not be included within the current test battery, the reliable impairment in 100% of five PCP-Iso cohorts now examined in our laboratory ([10, 12], and unpublished observations) suggests this deficit is also more robust than observed in 67% of 12 single-hit isolation cohorts. Accumulating behavioral data from the PCP-Iso dual-hit model therefore support an additive or cumulative stress hypothesis [65] as also observed for maternal separation [66] or neonatal AMPA/kainate receptor agonism [67] plus isolation rearing, and these newer models allow scope to investigate how complex interactions between early-life risk factors contribute to the neurobiology of neurodevelopmental disorders like schizophrenia. Models involving social manipulations like maternal separation and isolation rearing appear to have excellent construct validity for schizophrenia, since parental separation or loss, frequent relocation during adolescence, and social disadvantage or exclusion extending into later life are all risk factors [68]. While we acknowledge that developmental exposure to glutamate receptor ligands is less common [69–71] it appears the long-term consequences of such exposure in rodents as part of a dual-hit approach still afford good face validity. The availability of more robust preclinical models with which to test novel therapeutics has crucial relevance for drug discovery, but to our knowledge the pharmacological sensitivity of combined versus separate PCP and isolation models has not been directly compared before. Our principal finding is that dual-hit PCP-Iso are less sensitive to the 5-HT_6_ antagonist SB-399885, in terms of glutamate release from hippocampal slices, and this translates to reduced cognitive effect of SB-399885 in a NOD task. Reductions in hippocampal calbindin expression could potentially underlie both of these observations.

We recognize that the relevance of glutamate microsensor measurements is open to interpretation, because their invasive nature permits direct comparison with data from patients versus healthy controls. However, it is important to note that direct comparison between human and animal data are similarly lacking even in cases where the same MRS approach to study glutamatergic neurotransmission is used across species, due to requirements for general anesthesia in rodents. For compounds in the earlier phases of development glutamate microsensor measures in brain slices, or ultimately in freely moving animals, therefore provide a valuable direct indication of extracellular glutamate with high temporal resolution [24]. Basal extracellular glutamate levels in slices from control animals were similar to those obtained using alternative sensors [24], and components of the signal met several criteria for neuronal origin. These include sensitivity to KCl, α-latrotoxin, and TTX, with decreases to the latter in line with previous microsensor reports [72]. Neuronal glutamate sources in CA1 include CA3 Schaffer collateral synapses (onto basal and proximal apical pyramidal dendrites in strata oriens and radiatum) and entorhinal cortex temporoammonic projections (onto distal apical dendrites in stratum lacunosum-moleculare [73]). Precise replication of sensor placement between slices and animals was unattainable but typically within the region of apical dendrites and so receptive to both pathways. Reduced basal glutamate in isolates matches MRS findings [74] and mirrors the pattern of NOD impairments, consistent with dependence of this type of memory on glutamatergic neurotransmission within CA1 [33–36]. Although the consensus is that schizophrenia begins with hypofunction of NMDA receptors on GABAergic interneurons leading to disinhibition of pyramidal cells and excitotoxic damage to CA1 [75], MRS generally shows little hippocampal change in early schizophrenia [76]. Our NOD studies began at the accepted onset of adulthood, which is approximately 3 weeks after typical emergence of isolation-induced dopaminergic changes and hyperlocomotion [77]. Since our microsensor studies continued for almost 3 months after this first emergence it can be argued that current findings more closely mirror chronic disease where MRS can reveal decreased glutamate in patients [19], potentially due to reduced synaptic density [20] and VGLUT1 expression [21, 22]. Indeed, similar changes are reported in isolates within the time frame of our microsensor measurements [78, 79] and although we did not observe outright VGLUT deficits it is possible that subfield- and/or lamina-specific changes in expression were masked by analysis of whole homogenates. The altered ratio of VGLUT1:VGLUT2 may also contribute to the current isolation effect on basal glutamate, since these transporters respectively localize to synapses with low release probability that exhibit long-term potentiation or high release probability that exhibit long-term depression [80].

5-HT_6_ antagonists elevate hippocampal glutamate efflux in vivo [81] but the absence of similar effects when applied alone to synaptosomes [82] or slices is readily attributed to loss of 5-HT tone, while the lack of mGlu_7_ allosteric antagonist effect [83] is explained by relatively low affinity of glutamate for this receptor and consequent activation only during high-frequency firing [84]. Elevated extracellular K^+^ concentrations which occur during such discharge [85] are routinely used to induce neurotransmitter release ex vivo, and because KCl-evoked responses were unaffected by neurodevelopmental history, this stimulus appeared suitable for further examination of receptor-selective compounds. 5-HT_6_ blockade augments KCl-evoked glutamate release from slices in a TTX-sensitive manner [86] and we are the first to show this effect is attenuated in slices from PCP-Iso. We are also the first to reveal the potential functional correlates of this observation, since an SB-399885 dose capable of reversing isolation-induced NOD deficits was inactive in the combined neurodevelopmental model. While 5-HT_6_ antagonists’ effects on NOD ultimately depend on NMDA receptor-mediated glutamatergic mechanisms [48, 87, 88] and there is extensive colocalization of hippocampal 5-HT_6_ and VGLUT1 mRNA, any direct effect of excitatory Gα_s_ protein-coupled 5-HT_6_ receptors on principal neurons is played down by suggestions of little tonic 5-HT input to this population of 5-HT_6_ receptors [89]. Instead, GABAergic disinhibition is the main mechanism proposed to underlie 5-HT_6_ antagonist-induced glutamate release. The 5-HT_6_-expressing GABA interneurons appear to be largely calbindin-positive—not parvalbumin-positive (60 versus 5% mRNA co-localization, respectively [38]), which is consistent with preferential serotonergic innervation of the calbindin-positive interneurons that in turn arborize with proximal apical dendrites of pyramidal cells and mediate feedforward inhibition [37, 63]. In contrast, the mGlu_7_ antagonist-stimulated glutamate release that was maintained in PCP-Iso is likely to be largely independent of calbindin-positive interneurons, since Gα_i/o_ protein-coupled inhibitory mGlu_7_ autoreceptors and heteroreceptors are expressed on VIP-positive interneurons and appear to be preferentially found at synapses onto somatostatin-positive interneurons in stratum oriens that mediate feedback inhibition of distal pyramidal dendrites [90, 91]. It therefore appears plausible that dysfunction of calbindin-positive interneurons could account for attenuated glutamatergic and cognitive responses to SB-399885 and sparing of responses to MMPIP in PCP-Iso. The correlation between calbindin expression and NOD performance following SB-399885 does not confirm causality but at least appears consistent with our working hypothesis. We certainly saw no evidence for reduced 5-HT_6_ receptor expression in the dual-hit model, nor for reduced 5-HT innervation of the dorsal hippocampus that would influence 5-HT_6_ receptor tone. Admittedly, SERT expression or indicators of tonic 5-HT release in PCP-Iso remain unexplored, but any serotonergic dysfunction in schizophrenia [92], isolation-reared [93–95] or neonatal NMDA antagonist-treated rats [96, 97] appears far less extensive than that produced by intentional median raphe lesions that abolished 5-HT_6_ antagonists’ effect on NOD during dissociation of relevant neuroanatomical substrates [98].

There is evidence that some patients with schizophrenia do have reduced calbindin expression and a disordered pattern of calbindin interneurons in the hippocampus [99, 100, but see 101], while the VIP-positive interneurons that express mGlu_7_ remain unaffected in schizophrenia [102]. PCP-Iso therefore appear to have better relevance, in terms of face validity, for calbindin-deficient patient subgroups than single-hit isolation-reared rats or indeed other non-neurodevelopmental models. There has previously been some conflict whether reduced calcium-binding protein immunoreactivity in preclinical models and patients with schizophrenia reflects a selective posttranslational decrease in calcium-binding protein expression [103] or actual loss of that cell population [104]. Our PCP-Iso-induced calbindin decrease without any change in GAD_67_, VGAT, or 5-HT_6_ markers known to be present in the same cell types appears to support the former. Reduced calcium-binding protein expression would be expected to reduce interneuron firing [105], potentially preventing further 5-HT_6_ antagonist-mediated disinhibition. Future high-priority studies beyond scope of this manuscript should employ calbindin-deficient mice or RNA interference-mediated calbindin knockdown to test the hypothesis that a selective reduction in calbindin expression (of the order associated with schizophrenia) is actually sufficient to impact on 5-HT_6_ antagonist-mediated responses. It may become necessary to explore alternative explanations including alterations in frontal cortical dopamine, which is also important for NOD [106] and modulated by 5-HT_6_ antagonists [107] but as yet unexplored in PCP-Iso. Such alternatives may underlie the ability of cariprazine to reverse NOD deficits in PCP-Iso, since in vivo microdialysis shows this novel antipsychotic normalizes acute PCP-induced dopamine and glutamate efflux in the medial prefrontal cortex [108] and elevates dopamine efflux in the nucleus accumbens and hippocampus without having any effect on hippocampal glutamate [109]. On the basis of this latter observation, cariprazine would not be expected to influence the signal in our current microsensor studies and we therefore instead prioritized the inclusion of other putative procognitive drugs with established effects on glutamatergic neurotransmission within the hippocampus.

A diverse array of compounds, including 5-HT_6_ antagonists [42, 110–112], an mGlu_2/3_ agonist [49], NMDA receptor glycine modulatory site partial agonist [113], α4β2 and α7 nicotinic receptor agonists [114], donepezil [115], risperidone [47], and fluoxetine [116], all reverse “single-hit” isolation-induced NOD deficits. Some of these approaches are associated with clinical benefit in other psychiatric or neurodegenerative disorders but ultimately lack marked clinical benefit in schizophrenia [117–119]. For example, 5-HT_6_ antagonists appear to improve cognition in mild to moderate Alzheimer’s disease [120, 121] which interestingly, and consistent with our working hypothesis, does not appear to involve calbindin deficits in CA1 [122–124]. Sparse clinical data for cognitive effects of 5-HT_6_ antagonists in schizophrenia [125] together with anecdotal indications that most pharmaceutical companies have suspended trials in this area seem to imply that previous findings in normal rats and other models for this disorder may actually represent false positives. In support of this, it is worth noting the earlier failure of three independent groups to replicate procognitive effects of 5-HT_6_ antagonists in non-neurodevelopmental studies [126–128]. The current absence of any “gold standard” treatment for the cognitive impairment associated with schizophrenia makes it difficult to ascertain the true predictive validity of PCP-Iso. However, our previous findings with cariprazine, aripiprazole, and lamotrigine [12, 14], together with current observations with a 5-HT_6_ receptor antagonist, suggest wider adoption of PCP-Iso may aid more reliable preclinical evaluation of novel compounds to manage the symptoms of schizophrenia or even modulate disease onset. Any progress towards improved early distinction of promising from less suitable pharmacological approaches using the PCP-Iso model has clear relevance from streamlining drug discovery.

Paradoxically, 5-HT_6_ receptor agonists have similar cognitive effects to antagonists of the same receptor, so prolong NOD memory in normal animals [129] and reverse a variety of acute NMDA receptor antagonist-induced NOD deficits [130, 131, 132]. The underlying neurochemical mechanisms remain unclear, since 5-HT_6_ agonists actually facilitate GABAergic and inhibit glutamatergic neurotransmission in the hippocampus and cortex [53, 55, 82, 133]. Future neurochemical and cognitive studies in PCP-Iso should therefore examine the extent to which 5-HT_6_ receptor agonist-mediated responses might be perturbed by glutamatergic deficits and interneuron dysfunction in schizophrenia. The relationship between mGlu_7_ and memory is also complex, with the mGlu_7_-negative modulator MMPIP both impairing NOD in normal mice [58] and recently proposed as a putative antipsychotic due to its ability to reverse acute MK-801-induced hyperactivity and cognitive deficits in mouse NOD and rat spatial delayed alternation tests [56]. It has been suggested mGlu_7_ may represent a new treatment target for neurodevelopmental disorders [134], and current neurochemical and behavioral findings certainly appear to justify continued preclinical evaluation of mGlu_7_ allosteric antagonists against a broader variety of PCP-Iso-induced cognitive impairments. This is important since the NOD test of visual recognition memory performed here has translational relevance to only one of several cognitive domains impaired in schizophrenia [45]. Future studies should also assess the integrity of VIP-expressing interneurons in PCP-Iso to test our current working hypothesis that cells mediating the effects of mGlu_7_ receptor ligands would be spared in this animal model and therefore mirror findings in patients with schizophrenia [102]. In conclusion, this research highlights the importance of improved understanding for selection of appropriate preclinical models, and for better stratification of patient subpopulations to different drug treatments, especially in cases where disease neurobiology impacts upon the cells mediating pharmacological effects of potential therapeutics.

## Electronic Supplementary Material

ESM 1 (DOCX 291 kb)

ESM 2 (DOCX 21817 kb)

ESM 3 (DOCX 165 kb)
