# Supplementary material for: Calbindin Deficits May Underlie Dissociable Effects of 5-HT6 and mGlu7 Antagonists on Glutamate and Cognition in a Dual-Hit Neurodevelopmental Model for Schizophrenia
Source: Mol Neurobiol. 2020 Jun 12;57(8):3439–57. doi: 10.1007/s12035-020-01938-x (PMC7340678; doi:10.1007/s12035-020-01938-x)
Supplement: Supplementary file 1 — (DOCX 291 kb) [file 12035_2020_1938_MOESM1_ESM.docx]

**Supplementary** **Fig. 1** Confirmation of the neonatal PCP and isolation rearing-induced behavioral phenotype prior to tissue collection for microsensor and western blot analyses. Male Lister hooded rats that received saline (1 ml/kg s.c.; Veh) or PCP (10 mg/kg) on PND 7, 9 and 11 were housed in groups (Gr) or isolation (Iso) from weaning on PND 21 (n = 15-18 per treatment-housing combination), then underwent tests of locomotor activity (PND 56-58), novel object discrimination (NOD, PND 57-59) and pre-pulse inhibition (PPI) of the acoustic startle response (PND 63). Data are mean ± SEM (**a**-**b**) number of infra-red beam breaks for (**a**) the timecourse and (**b**) total ambulatory activity in a novel arena, (**c**-**d**) time spent exploring (**c**) two identical objects during the NOD familiarization trial and (**d**) novel and familiar objects during the NOD choice trial 2 h later, as well as (**e**) the choice trial discrimination ratio (time exploring novel/total choice trial object exploration) and (**f**) percent inhibition during PPI testing. The (**a**) timecourse of ambulatory activity showed a time x housing interaction (*P*<0.001) and (**b**) total ambulation was influenced by treatment (*P*<0.05) and housing (*P*<0.05). Ambulation was higher in PCP-Iso (**a**) at multiple timepoints and (**b**) in total. Object exploration in (**c**) the familiarization trial was unaffected by treatment or housing but during (**d**) the choice trial showed a main effect of object (*P*<0.001) and an object x housing interaction (*P*<0.001), with intact discrimination in Veh-Gr and PCP-Gr and impaired memory in Veh-Iso and PCP-Iso. This pattern was mirrored in (**e**) the choice trial discrimination ratio, which showed a main effect of housing (*P*<0.0001) and was reduced in Veh-Iso and PCP-Iso. PPI testing (**f**) showed a main effect of pre-pulse volume (*P*<0.001) although the pre-pulse x housing interaction just failed to reach significance (*P*=0.057). ^†^/+/#*P*<0.05 PCP-Iso versus Veh-Gr/PCP-Gr/Veh-Iso and ****P*<0.001; *****P*<0.0001 versus the familiar object in the same rats (two-way ANOVA (**b**, **e**) or three-way repeated measures ANOVA (**a**, **c**-**d**, **f**) with Tukey (**a**-**b**, **e**) or Sidak (**c**-**d**) post-hoc).
