# Supplementary material for: Calbindin Deficits May Underlie Dissociable Effects of 5-HT6 and mGlu7 Antagonists on Glutamate and Cognition in a Dual-Hit Neurodevelopmental Model for Schizophrenia
Source: Mol Neurobiol. 2020 Jun 12;57(8):3439–57. doi: 10.1007/s12035-020-01938-x (PMC7340678; doi:10.1007/s12035-020-01938-x)
Supplement: Supplementary file 2 — (DOCX 21817 kb) [file 12035_2020_1938_MOESM2_ESM.docx]

**Supplementary** **Fig. 2** Effect of neonatal PCP and isolation rearing on hippocampal expression of glutamatergic and GABAergic markers, 5-HT_6_ and mGlu_7_ receptors. Representative western blots and bar graphs of mean ± SEM expression of (**a**) VGLUT1, (**b**) VGLUT2, (**c**) VGLUT3, (**d**) EAAT1, (**e**) EAAT2, (**f**) EAAT3, (**g**) GAD_67_, (**h**) VGAT, (**i**) 5-HT_6_ and (**j**) mGlu_7_ as percentages of GAPDH. Male Lister hooded rats that received saline (1 ml/kg s.c.; Veh) or PCP (10 mg/kg) on PND 7, 9 and 11 were housed in groups (Gr) or isolation (Iso) from weaning on PND 21, with tissue collection on PND 64 (n = 8-10 per treatment-housing combination). There was a subtype x treatment x housing interaction for VGLUT expression (*P*<0.05) and a treatment x housing interaction for EAAT2 expression (*P*<0.05), which was lower in PCP-Iso than Veh-Iso. There was also a treatment x housing interaction for 5-HT_6_ expression (*P*<0.05) but between-group differences did not reach post-hoc significance. #*P*<0.05 versus Veh-Iso (two-way ANOVA with Tukey post-hoc).
