# Supplementary material for: Calbindin Deficits May Underlie Dissociable Effects of 5-HT6 and mGlu7 Antagonists on Glutamate and Cognition in a Dual-Hit Neurodevelopmental Model for Schizophrenia
Source: Mol Neurobiol. 2020 Jun 12;57(8):3439–57. doi: 10.1007/s12035-020-01938-x (PMC7340678; doi:10.1007/s12035-020-01938-x)
Supplement: Supplementary file 3 — (DOCX 165 kb) [file 12035_2020_1938_MOESM3_ESM.docx]

**Supplementary** **Fig. 3** Effect of neonatal PCP and isolation rearing on hippocampal ratios of vesicular glutamate transporter expression. Mean ± SEM expression of VGLUT1 as (**a**) the ratio of VGLUT1:VGLUT2 and (**b**) a proportion of total VGLUT1-3 expression. Male Lister hooded rats that received saline (1 ml/kg s.c.; Veh) or PCP (10 mg/kg) on PND 7, 9 and 11 were housed in groups (Gr) or isolation (Iso) from weaning on PND 21, with tissue collection on PND 64 (n = 8-10 per treatment-housing combination). There were treatment x housing interactions for both indices of VGLUT1 expression (*P*<0.05), which were lower in PCP-Iso than PCP-Gr. +*P*<0.05 versus PCP-Gr (two-way ANOVA with Tukey post-hoc).
